# Competing-risk analysis of death and dialysis initiation among elderly (≥80 years) newly referred to nephrologists: a French prospective study

**DOI:** 10.1186/1471-2369-14-103

**Published:** 2013-05-07

**Authors:** Bernadette Faller, Jean-Baptiste Beuscart, Luc Frimat

**Affiliations:** 1 Department of Nephrology, Hôpital Louis Pasteur, Colmar, France; 2 Department of biostatistics, EA 2694, UDSL, Lille, France; Geriatric Department, University Hospital, Lille, France; 3 Department of Nephrology, University Hospital, Vandœuvre-lès-Nancy, France; 4 Nancy University, P. Verlaine Metz University, and Paris Descartes University, EA 4360 Apemac, Nancy, France

**Keywords:** Chronic kidney disease, Renal replacement therapy, Conservative management, Elderly, Competing-risk analysis

## Abstract

**Background:**

Reasons underlying dialysis decision-making in Octogenarians and Nonagenarians have not been further explored in prospective studies.

**Methods:**

This regional, multicentre, non-interventional and prospective study was aimed to describe characteristics and quality of life (QoL) of elderly (≥80 years of age) with advanced chronic kidney disease (stage 3b-5 CKD) newly referred to nephrologists. Predictive factors of death and dialysis initiation were also assessed using competing-risk analyses.

**Results:**

All 155 included patients had an estimated glomerular filtration rate (eGFR) below 45 ml/min/1.73 m^2^. Most patients had a non anaemic haemoglobin level (Hb) with no iron deficiency, and normal calcium and phosphate levels. They were well-fed and had a normal cognitive function and a good QoL. The 3-year probabilities of death and dialysis initiation reached 27% and 11%, respectively. The leading causes of death were cardiovascular (32%), cachexia (18%), cancer (9%), infection (3%), trauma (3%), dementia (3%), and unknown (32%). The reasons for dialysis initiation were based on uncontrolled biological abnormalities, such as hyperkalemia or acidosis (71%), uncontrolled digestive disorders (35%), uncontrolled pulmonary or peripheral oedema (29%), and uncontrolled malnutrition (12%). No patients with acute congestive heart failure or cancer initiated dialysis. Predictors of death found in both multivariate regression models (Cox and Fine & Gray) included acute congestive heart failure, age, any walking impairment and Hb <10 g/dL. Regarding dialysis initiation, eGFR <23 mL/min/1.73 m^2^ was the only predictor found in the Cox multivariate regression model whereas eGFR <23 mL/min/1.73 m^2^ and diastolic blood pressure were both independently associated with dialysis initiation in the Fine & Gray analysis. Such findings suggested that death and dialysis were independent events.

**Conclusions:**

Octogenarians and Nonagenarians newly referred to nephrologists by general practitioners were highly selected patients, without any symptoms of the common geriatric syndrome. In this population, nephrologists’ dialysis decision was based exclusively on uremic criteria.

## Background

Nephrologists are increasingly confronted with an elderly population of patients who have a large number of co-morbid conditions requiring ongoing care [1]. Indeed elderly patients with chronic kidney disease (CKD) experience a high rate of complications, such as atherosclerotic cardiovascular disease, congestive heart failure, diabetes mellitus, cognitive and functional impairment, and CKD-related complications including anaemia [1-5]. CKD is present at a high rate in the population of elderly aged 70 and over, affecting about one person out of three in France, but only a few of them present clinically relevant markers requiring appropriate care [6]. CKD at a moderate and more severe stage defined by an estimated glomerular filtration rate (eGFR) level <60 mL/min/1.73 m^2^ affects about 38% of subjects over 70 years in the United States [7]. Additionally, overall incidence of end-stage renal disease (ESRD) is still increasing in French elderly aged 85 years or older, and especially in diabetics [6].

Old age is no longer seen as an absolute contraindication of dialysis in most industrialized countries [6,8-11]. In France, about 3 individuals out of 1000 elderly over 75 years benefit from a renal replacement therapy, dialysis or renal transplantation, with a significant increase of dialysis prevalence [6]. However, deciding whether or not dialysis can offer a substantial prolongation of life expectancy with an acceptable quality of life (QoL) among pre-ESRD (end-stage renal disease) Octogenarians is still a difficult task for nephrologists [5,8-11]. Advanced dementia and severe neurological sequels of stroke were shown as the conditions underlying the nephrologists’ decision-making not to provide dialysis in elderly patients [12].

Recent studies suggested that dialysis provided a survival advantage compared to conservative management for most of stages 4-5 CKD patients over the age of 75 [13-15]. However, this advantage was lost for patients with multiple co-morbidities and ischemic heart disease [13-15]. Particularly, Demoulin *et al*. raised the question of the proportion of patients with stage 4 CKD unnecessarily prepared for dialysis in a cohort of 386 patients including some octogenarians [15].

In a context of a lack of prospective studies exploring dialysis decision-making in Octogenarians and Nonagenarians, we implemented the OPAIR (étude Observationnelle des Patients Agés Insuffisants Rénaux) study in 2007. This trial was aimed to: 1) describe the characteristics of French very elderly pre-ESRD patients newly referred to nephrologists, and their QoL; 2) evaluate death, dialysis initiation, and their predictive factors. Additionally, these two clinical outcomes were incorporated into competing-risk analyses used, to the best of our knowledge, for the first time in elderly [16].

## Methods

This was a regional, multicentre, non-interventional and prospective study conducted in a cohort of very elderly pre-ESRD patients not on dialysis recruited by 20 nephrology centres in France.

### Study population

Patients were included between April 2007 and April 2008 if they were: incident patients newly referred to nephrologists or prevalent patients with a nephrology follow-up less than nine months (started after July 1^st^, 2006), ≥80 years of age, not on dialysis, and with serum creatinine >170 μmol/L for men and >150 μmol/L for women to guarantee the inclusion of CKD patients with a high risk of disease progression. Nephrologists provided prognosis information to patients and/or families when discussing inclusion or exclusion for dialysis during several consultations. The acceptance of dialysis if necessary was not therefore an inclusion criterion.

Patients with a planned dialysis in the next three months were excluded from the study.

**Figure 1:**
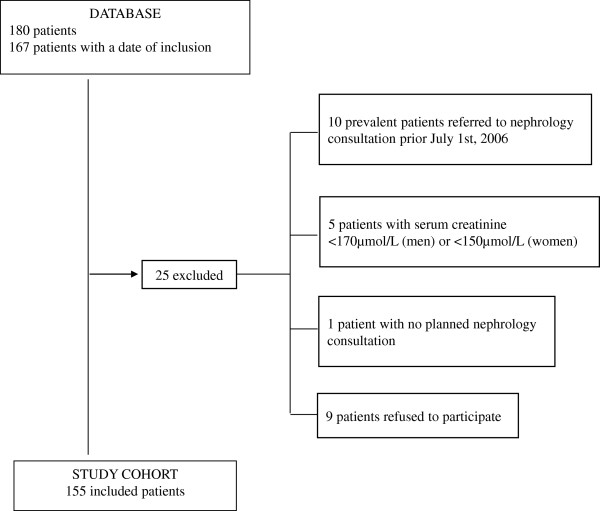
The OPAIR study flow chart.

Among the 180 eligible patients registered in the database, 25 subjects were excluded for a final study population of 155 subjects, as outlined in Figure 1.

### Outcomes

After inclusion, the study follow-up was designed for 2 years. However, frequency of nephrology consultations was freely determined by the nephrologists. Finally, all the patients were tracked until the occurrence of death, dialysis initiation, lost of follow-up, or the end of the study (December 31^st^, 2010), whichever came first.

### Data collection

For each inclusion of a new patient, a standardized form was prospectively completed. At the time of study entry, extensive clinical data were collected, including demographics, lifestyle, physical examination, medical history, co-morbid conditions, and biochemical data. Cognitive status was screened using the Mini Mental State Examination (MMSE). This 30-point questionnaire was administered by trained staff to ensure reliability of data collection [17]. A mean MMSE score greater than or equal to 24 points (out of 30) illustrated a normal cognitive function for patients. Health-related QoL was assessed using the validated Medical Outcomes Study 36-Item Short-Form Health Survey (SF-36) across eight dimensions (physical functioning, role-physical, bodily pain, general health, vitality, social functioning, role emotional, mental health) using 36 items [18]. The SF36-questionnaire was fully explained to each patient, and then the forms were filled out by patients themselves. Two summary scores were obtained: physical health score by aggregating physical functioning, role-physical, bodily pain and general health, and the mental health score by aggregating vitality, social functioning, role emotional, and mental health. The SF-36 produced a score on a 0–100 scale for each dimension and each summary measure, a low score indicating a poor health status.

During the study, occurrence of death and dialysis initiation was reported. As the study was non interventional, the participating nephrologists were free to plan follow-up care visits according to their usual practice.

Data about prior decision to offer dialysis or conservative care were not collected at the start of the study.

### Ethics statement

No French ethics committee oversight was required as the design of the study was strictly observational [19].

Each patient was informed about the aim and the course of the study in agreement with French regulations concerning observational studies [19]. After reading the patient information sheet, physicians obtained a verbal consent from each patient. All patients had capacity to consent or not. A written consent was not required for this kind of study based on the strict respect of usual medical practice and physician-patient relationship.

Anonymity was guaranteed. Data processing was under the modified law of January 6^th^, 1978 relating to the protection of data subjects regarding the processing of personal data.

### Statistical analysis

We estimated that a sample size of 200 patients would be sufficient to detect 25% of patients on dialysis at the end of the 24-month follow-up period with a precision of 6% and a 95% confidence interval (CI).

Baseline characteristics were described as means and standard deviations (SD) for continuous variables, and frequencies and proportions for categorical variables.

In survival analysis, the two outcomes of interest were death and dialysis initiation. They were considered as competing risks, whereas other events were censored. A competing risk is an event which either hinders observation of the event of interest, or modifies its probability of occurrence [20,21]. When competing risks are present, analysis of time-to-event data requires the adoption of specific methods, which may in turn influence the results and their interpretation [16,20,22].

The crude cumulative incidence functions were estimated for death and dialysis initiation using the method of Kalbfleisch and Prentice [21,23]. As recommended, the duration shown on the cumulative incidence curves was extended to 3 years because only 10% of the patients were still under follow-up at this time [24].

Bivariate analyses were performed using the Cox proportional hazard regression model on the cause-specific hazard of death and dialysis initiation, and the Fine and Gray regression model on the sub-distribution hazard of death and dialysis initiation [22,25].

We used four multivariate survival regression models on the whole cohort: i) a Cox proportional hazard regression model on the cause-specific hazard of death, ii) a Cox proportional hazard regression model on the cause-specific hazard of dialysis initiation, iii) a Fine & Gray regression model on the sub-distribution hazard of death, iv) a Fine & Gray regression model on the sub-distribution hazard of dialysis initiation [22]. The Fine & Gray model provides complementary competing-risk data to the Cox proportional hazards model by considering the sub-distribution hazard [16].

The STROBE guidelines recommend that cohort studies report on the amount of missing data and the method used to handle missing data [26]. For covariates with missing values, we obtained values by multiple imputations using the MICE package as recommended for the Cox proportional hazard model analysis [27]. This was achieved through regression switching imputation using linear or logistic regression models depending on the nature of the incomplete covariate fitted [27,28]. This procedure was repeated five times to obtain five draws for each missing value in five distinct datasets.

Covariates were selected in the multivariate analysis using a stepwise procedure adapted to multiple imputation methodology [29]. It was not possible to include the covariates “Acute Congestive Heart Failure” and “Cancer” in bivariate and multivariate regression analyses on the cause-specific hazard and sub-distribution hazard of dialysis initiation, because no dialysis events occurred for patients with such characteristics (no convergence of models). Rubin’s approach was adopted, whereby the coefficients and variances obtained with the final model on each imputed dataset were averaged by taking into account the intra-variance of the model and the inter-variance between the imputed datasets [30].

Additionally, the Cox model implicitly assumes a log-linear model for the continuous variables, i.e. the risk ratio for the ages of 20 to 30 years is the same as that for the ages of 70 to 80 years. The log-linear assumption was assessed using the Martingale residuals [31]. Since the log-linearity assumption was violated for haemoglobin and eGFR, they were transformed into categorical variables. The cut-off values were identified as follows: i) with graphic investigations using Martingale residual plots; ii) with maximization of the Gray test; and iii) on the basis of medical expertise and consensus.

All statistical management was performed using the R statistical programming language and computing environment with survival, cmprsk, and MICE packages [32].

## Results

After inclusion visit (visit 1: n=155), most of the patients received further nephrology consultations (visit 2: n=137, visit 3: n=125, visit 4: n=107, visit 5: n=88, visit 6: n=62, visit 7: n=43, visit 8: n=30, visit 9: n=18, visit 10: n=13, visit 11: n=6, visit 12: n=1, visit 13: n=1, visit 14: n=1). The mean duration of the follow-up was 24.7 months (inter-quartile range: 17.9, 26.9).

### Patient characteristics

Baseline characteristics of the cohort are shown in Table 1. Patients were octogenarians and nonagenarians with a median age of 84 years at study entry. Most of them lived at home, in couple, and walk independently (Table 1). Among patients with handicap (n=43), 40% of them had hearing loss, 16% severe visual impairment, 14% dementia, 7% hemiplegia, and 37% other disorders.

**Table 1:** Demographic and baseline characteristics

**Characteristics**			**N=155**	**Missing data ** **n (%)**
**Demography**	Age, years		85±3.8	0 (0.0%)
	Sex ratio		1:1	0 (0.0%)
	Social data, n (%)			36 (23.4%)
		*Live at home*	133 (90.5%)	
		*Live in couple*	63 (51.6%)	
**Smoking status, n (%)**				19 (12.3%)
	No smoking		94 (69.1%)	
	Former or current smoking		42 (30.9%)	
**Clinical**	BMI, kg/m^2^		26.1±5.2	21 (13.6%)
	SBP, mm Hg		142.9±20.5	0 (0.0%)
	DBP, mm Hg		73.5±10.6	0 (0.0%)
	Walking			10 (6.5%)
		*Total disability*	4 (2.8%)	
		*Need assistance*	44 (30.3%)	
		*Walk independently*	97 (66.9%)	
**Handicap, n (%)**			43 (30.3%)	13 (8.4%)
**Peripheral oedema, n (%)**			58 (38.2%)	3 (1.9%)
**Aetiology of renal disease, n (%)**				1 (0.6%)
	Vascular nephropathy*		53 (34.4%)	
	Diabetic nephropathy		11 (7.2%)	
	Tubulointerstitial nephritis		11 (7.2%)	
	Glomerulonephritis		3 (1.9%)	
	Undefined aetiology		59 (38.3%)	
	Other		17 (11.0%)	
Stages CKD**, n (%)				0 (0.0%)
	Stage 3b CKD		35 (22.3%)	
	Stage 4 CKD		105 (67.8%)	
	Stage 5 CKD		15 (9.9%)	
**Cognitive function, MMSE score**			26.0±4.2	34 (22.1%)
**Quality of life, SF-36 score**				49 (31.8%)
	Physical health summary score		35.5±8.9	
	Mental health summary score		45.2±10.5	
**Laboratory tests**	Serum creatinine, mg/dL		2.5±0.8	0 (0.0%)
	eGFR, ml/min/1.73m^2^		24.3±6.7	0 (0.0%)
	Blood urea, mmol/L		14.3±10.0	31 (20.1%)
	Albumin, g/dL		3.9±0.5	79 (51.3%)
	CRP, mg/dL		0.9±1.0	70 (45.5%)
	Proteinuria, g/24 hours		0.98±1.79	83 (53.9%)
	Cholesterol, mmol/L		5.0±1.1	74 (48.1%)
	HDL, mmol/L		1.7±2.0	93 (60.4%)
	LDL, mmol/L		3.2±3.2	93 (60.4%)
	Triglycerides, mmol/L		1.5±0.8	74 (48.1%)
	Calcium, mmol/L		2.3±0.2	27 (17.5%)
	Phosphate, mg/dL		3.8±0.9	36 (23.4%)
	Haemoglobin, g/dL		11.7±1.7	15 (9.7%)
	Haematocrit, %		35.6±5.2	23 (14.9%)
	Ferritin, ng/mL		226.3±325.0	90 (58.4%)
	TSAT, %		23.5±9.2	118 (77.6%)

Data expressed as mean ± standard deviation or n (%) when appropriate.

* including HTA; ** according to Modified diet in renal disease (MDRD’s formula).

BMI=body mass index, SBP=systolic blood pressure, DBP=diastolic blood pressure, CKD=chronic kidney disease, MMSE=mini mental state examination, SF-36=medical outcomes study 36-item short-form health survey, eGFR=estimated glomerular filtration rate, CRP=C-reactive protein, HDL=high density lipoprotein, LDL=low density lipoprotein, TSAT=transferrin saturation.

Unknown causes and vascular nephropathies were the most frequent aetiologies of renal disease (Table 1). CKD was severe as outlined in Table 1. All patients had an eGFR below 45 ml/min/1.73 m^2^ [according to the Modified Diet in Renal Disease (MDRD)’s formula]. Few patients had undergone renal biopsies (2%) or nephrectomy (6.5%).

All laboratory results are presented in Table 1. Most patients had a non anaemic haemoglobin level (Hb ≥11 g/dL: 66%) with no iron deficiency [ferritin level ≥100 ng/mL: 59%; transferrin saturation (TSAT) ≥20%: 63%]. Regarding inflammatory status of patients, 70% of patients had a C-reactive protein (CRP) level <10 μg/dL.

Most of patients were non smokers (Table 1) and only 16% of patients had a history of obesity. Regarding lipid disorders, 58% of patients had none, 33% hypercholesterolemia and 9% hypertriglyceridemia. The majority of patients were non diabetic (67%) as outlined in Table 2.

**Table 2:** Summary of co-morbidities (N=155)

**Co-morbidities, n (%)**	**N=155**
Diabetes	51 (33.4%)
Arrhythmia	58 (37.7%)
Coronary artery disease	33 (21.6%)
Congestive heart failure*	22 (14.3%)
Valvular heart disease	18 (11.7%)
Peripheral vascular disease	27 (17.6%)
Stroke	16 (10.3%)
Neoplasia (<5 years)	21 (13.7%)
Respiratory insufficiency	18 (11.7%)
Continuous oxygen therapy	1 (0.6%)
Cirrhosis	2 (1.3%)

* including 19 episodes of acute congestive heart failure.

Regarding cognitive performance, 81% of men and 71% of women had a normal cognition as their mean MMSE score was greater than or equal to 24. According to the age of patients, 77% of patients aged <90 years and 67% of patients ≥90 years had a normal cognitive function (MMSE score ≥24).

**Figure 2:**
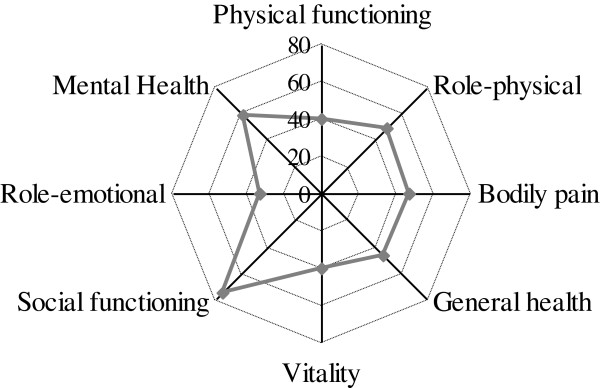
**Quality of life at inclusion (using SF-36 questionnaire).** Medians are represented.

Data concerning QoL at inclusion using SF-36 questionnaire are presented as median scores in Figure 2. Their physical and mental health summary scores are presented in Table 1.

### Outcomes

A total of 83 patients were prematurely withdrawn for the following reasons: 18 patients with no further planned nephrology consultation, 17 patients initiated dialysis, 41 patients died and 7 patients were lost to follow-up. Finally, 72 patients were still alive at the end of the study.

**Figure 3:**
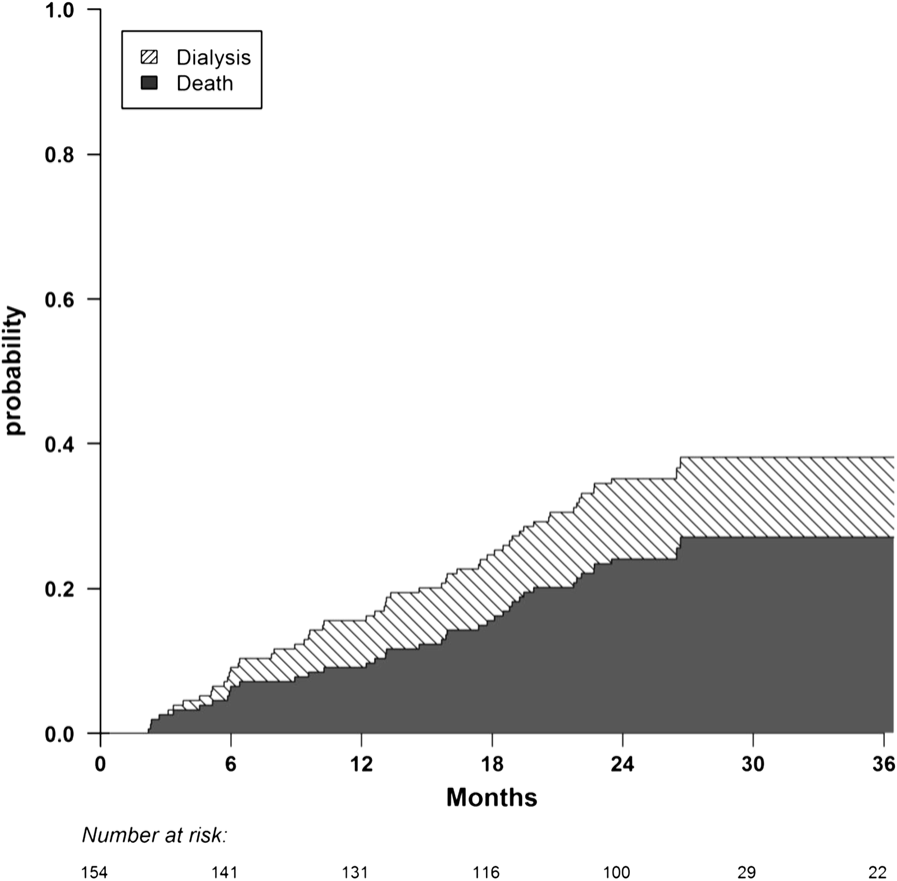
**Cumulative incidence of death and dialysis initiation during follow-up.** Cumulative incidences are stacked.

The 3-year probabilities of death and dialysis initiation reached 27% and 11% respectively, as shown in Figure 3. The leading causes of death were cardiovascular (32%), cachexia (18%), cancer (9%), infection (3%), trauma (3%), dementia (3%), and unknown (32%).

No dialysis initiation was observed among 19 patients with acute congestive heart failure at inclusion, and 21 patients with a history of cancer within the last 5 years before inclusion. Thus a total of 40 patients (26%) were withdrawn from dialysis only because of these antecedents.

Regarding the 17 patients who initiated dialysis, they underwent in majority hemodialysis (n=12, 71%) or peritoneal dialysis (n=5, 29%). Among hemodialysis patients, 7 patients started treatment with an arteriovenous fistula and 5 patients with a venous catheter. The reasons for dialysis initiation were based on uncontrolled biological abnormalities, such as hyperkalemia or acidosis (n=12, 71%), uncontrolled digestive disorders (n=6, 35%), uncontrolled pulmonary oedema or peripheral (n=5, 29%), and malnutrition (n=2, 12%). The delay before initiation of dialysis was 12±6 months. The mean age of these patients was 84.9±2.9 years when dialysis therapy was started; overall values ranged from 80.5 to 89.3 years. The median eGFR was 10.3 (interquartile range: 7.8-14.4) mL/min/1.73 m^2^; overall values ranged from 6.0 to 19.4 mL/min/1.73 m^2^. Among them, 7 patients died before the end of the study. For these dialysis patients, one-year and two-year survival rates were 64.7% and 58.8%, respectively. Additionally, 3 patients underwent fistula creation without receiving dialysis.

### Competing-risk analysis of death and dialysis initiation

Variables associated with death using Cox bivariate regression analysis were: handicap, acute congestive heart failure, peripheral oedema, age, MMSE score, walking impairment, and haemoglobin level (Hb) <10 g/dL (data not shown). In the Cox multivariate regression analysis, acute congestive heart failure, age, walking impairment and Hb<10 g/dL were independently associated with death as shown in Table 3. All predictors of death in the Fine & Gray model were those found in the Cox model (Table 3). No other variables as those identified in the Cox model were significant in the Fine & Gray analysis.

**Table 3:** Competing-risk models of variables associated with death and dialysis initiation (multivariate regression Cox and Fine & Gray models)

**Outcomes**	**Predictive variables**	**Cox analysis**		**Fine & Gray analysis**	
		**RR**	**CI**	**RR**	**CI**
**Death**	Acute congestive heart failure	2.62	[1.17 - 5.89]	2.86	[1.31 - 6.26]
	Age (years)	1.09	[1.01 - 1.18]	1.09	[1.01 - 1.18]
	Walking impairment	2.11	[1.00 - 4.43]	2.17	[1.05 - 4.49]
	Hb <10 g/dL	3.73	[1.74 - 7.99]	3.85	[1.77 - 8.39]
**Dialysis initiation**	DBP (per cm Hg)	1.03	[0.99 - 1.07]	1.03	[1.01 - 1.06]
	eGFR <23 mL/min/1.73m^2^	12.95	[2.93 - 57.18]	13.37	[3.04 - 58.69]
	Hb <10 g/dL	0.49	[0.06 – 3.75]	0.32	[0.04 – 2.57]

RR=risk ratio, CI=confidence interval, Hb=haemoglobin, DBP=diastolic blood pressure, eGFR=estimated glomerular filtration rate.

Variables associated with dialysis initiation using Cox bivariate regression analysis were: serum creatinine level, eGFR <23 mL/min/1.73 m^2^, systolic and diastolic blood pressure (DBP, data not shown). In the Cox multivariate regression analysis, eGFR <23 mL/min/1.73 m^2^ was the only variable independently associated with dialysis initiation as shown in Table 3. However, in the Fine & Gray analysis, eGFR <23 mL/min/1.73 m^2^ and DBP were both independently associated with dialysis initiation as shown in Table 3.

**Figure 4:**
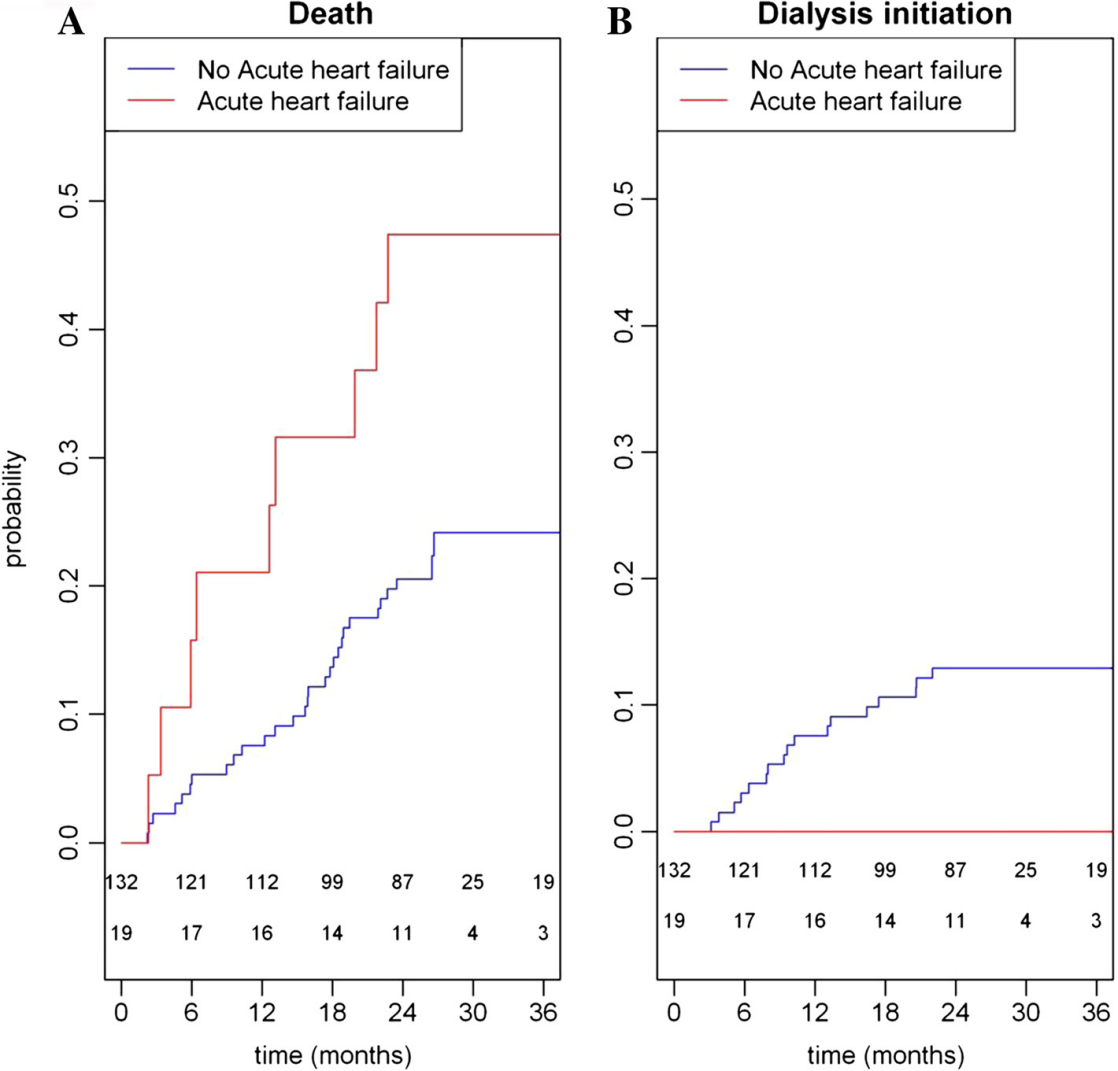
Cumulative incidences of death (panel A) and dialysis initiation (panel B) for patients with acute heart failure (red) and other patients (blue).

Patients with acute congestive heart failure were 2.62-fold more likely to die than others (Figure 4 - panel A). In the other hand, they were not offered dialysis treatment (Figure 4 - panel B). Consequently, acute congestive heart failure was a comorbid condition both predictor of death and dialysis initiation. Moreover, no other predictor of death was found as predictor of dialysis initiation in both models (Cox and Fine &Gray) (Table 3), suggesting that the 2 clinical outcomes were independent for patients without acute congestive heart failure.

## Discussion

The major strength of the OPAIR study is its prospective and observational design with measurements of QoL and cognitive function. We focused on CKD management in Octogenarians and Nonagenarians before dialysis decision-making in addition to previous studies devoted to elderly including patients starting dialysis [9,10], and those conducted in general population [4,33,34]. We provided useful information on a very elderly pre-ESRD French population newly referred to nephrologists, and dialysis decision-making in usual clinical practice in this population. Death and dialysis initiation were found to be independent clinical outcomes, except for acute congestive heart failure patients. Finally, we highlighted that the patient’s age had no impact on dialysis decision as nephrologists seem to use similar acceptance criteria for dialysis in very elderly as those in younger subjects.

The OPAIR study prospectively profiled the clinical characteristics of very elderly French patients with an advanced CKD newly referred to nephrologists. Regarding kidney disease, most of them had a severely impaired renal function, and should potentially progress to ESRD requiring a renal therapy replacement or die. Nephrologists considered going into aetiology of renal disease in patients (2% biopsies) of no importance, suggesting a probabilistic aetiology. However, CKD-related complications were meticulously controlled; most of them had a haemoglobin level over 11 g/dL, and normal calcium and phosphate levels. Among OPAIR participants, the 3-year probabilities of death and dialysis initiation amounted to 27% and 11%, respectively. Such findings suggest that participants were 2.5-fold more likely to die than to progress to ESRD requiring dialysis. Our results were relatively consistent with those found in previous studies with disparate settings showing death, largely cardiovascular, as the most common outcome in CKD patients rather than progression to advanced stages of CKD, even among patients with stage 4 CKD, and especially true in elderly [4,8-10,13,15,33,35].

This specific geriatric population was relatively healthy. Included patients had moderate co-morbidities, a preserved cognitive function and no deterioration of QoL. Their physical and mental health summary scores illustrated indeed a good health status despite their age and were close to those of younger elderly (≥75 years) in general population [36]. They were also well-fed. With regard to management of very elderly (≥80 years) with renal disease, there was therefore an indication bias in the selection process on behalf of GPs towards referring patients for nephrology consultation in the OPAIR study. GPs, consciously or unconsciously, had restricted access to patients who could potentially require a dialysis, based on renal criteria and good health. Indeed most patients referred to nephrologists were neither elderly institution residents, nor malnourished frail elderly with geriatric syndrome. Such results outlined that the clinical profile of these very elderly pre-ESRD patients did not fit to the profile of geriatric patients.

Congestive heart failure or a history of cancer had a significant impact on access to dialysis. No included patients with acute congestive heart failure underwent dialysis. This condition, by contrast, was found as a strong predictor of death. Additionally, no patients with a history of cancer underwent dialysis, whereas no increased risk of death from cancer was found. Thus 26% of patients with one of these two antecedents, easily recognized by clinicians, were not offered dialysis. The observational nature of data cannot explain the process that has led to a lack of dialysis for these patients. Several hypotheses can be advanced. First, patients with congestive heart failure had a higher risk of death, and therefore died maybe before requiring dialysis. Second, nephrologists decided not to offer dialysis for fear of worsening the quality of life without improving survival in such patients. Indeed a recent study has suggested that there is no benefit in terms of survival for patients with cardiovascular co-morbidities [13]. Third, patients with heart failure or a history of cancer were more able to decline to undergo dialysis. As none of these patients underwent dialysis, it is likely that these three hypotheses were combined. Such a result suggests that nephrologists have introduced a dialysis indication bias in patients with congestive heart failure or a history of cancer.

Finally, competing risk-analyses in the OPAIR study focused on a geriatric population newly referred to nephrologists with a good health status, despite their old age. As both competing-risk analyses (Cox, Fine and Gray) enable us to conclude that probabilities of death and dialysis initiation were independent in this specific geriatric population, they reinforce the identification of both indication bias in the selection process on behalf of GPs and nephrologists. The nephrologists’ dialysis decision-making was based exclusively on renal criteria as we found that eGFR <23 mL/min/1.73 m^2^ was the main predictive factor of dialysis initiation. In the opposite, eGFR <23 ml/min/1.73 m^2^ was not a predictor of death in competing-risk frameworks of the OPAIR study. Our findings suggest that predictors of death, such as age, walking impairment and anaemia (Hb <10 g/dL) in very elderly pre-ESRD patients newly referred to nephrologists seem to be features of physiological ageing process.

It is well-known that patients with severe co-morbidities may not always benefit from dialysis [15,37,38]. Of note, the prevalence of diabetes and cardiovascular diseases in French 2009 incident dialysis patients, 41% and 57% respectively, was significantly higher than in the largely older OPAIR patients [39]. Otherwise, in a vignette study, a consensus seemed to exist across GPs, non nephrology specialists and nephrologists about recognizing that dialysis was not appropriate for elderly patients under some circumstances such as terminal cancer, but still on debate about other co-morbidities, such as mild shortness of breath, diabetes and mild cognitive impairments [37]. At the opposite, younger CKD patients are usually referred to nephrologists on the basis of the level of renal function whatever their co-morbid conditions, QoL and cognitive function are. Finally, our study reinforce recommendations, emphasizing the importance of referral to specialist nephrology services largely for CKD patients on the basis of glomerular filtration rate (stage 4-5) or high levels of proteinuria [40].

In clinical practice, it is usual to start dialysis aimed to potentially improve among CKD patients less than 80 years of age and stop it if no improvement of patient’s condition is observed. At the opposite, decision to initiate dialysis among patients aged 80 years and over is based on medical considerations with strong arguments that dialysis will offer sufficient survival benefit taking into account the treatment burden of dialysis, in the best interest of the patients. In clinical practice, the appropriate decision-making in CKD elderly is based on a subtle balance between overtreatment by initiating dialysis earlier than may be necessary or maintaining dialysis with no increase in life expectancy or no improvement of QoL, and undertreatment involving late referrals to nephrologists and unplanned dialysis that leads to severely impaired QoL [11,41]. Among more than 1.8 million adults in a community-based cohort, untreated CKD among adults aged 75 years or older with baseline eGFR of 15 to 29 mL/min/1.73 m^2^ was approximately 2- to 10-fold more common than CKD treated by dialysis [42]. After dialysis decision-making, primary concerns of nephrologists are focused on the right moment to adequately prepare patients for dialysis and initiate dialysis in elderly. Other challenge for nephrologists treating very elderly population is posed by the choice of access and the timing of its creation. Recently, Hiremath *et al*. have suggested that the optimal strategy in elderly with stage 4 CKD, excluding those with a proteinuric diabetic nephropathy, should be to wait and start with a central venous catheter when required followed by an arteriovenous fistula creation, whereas most guidelines have recommended assessment of patients for access creation at stage 4 CKD [43-45].

The latest updated guidelines suggested that the timing of dialysis therapy initiation in older patients with advanced CKD should remain focused on individualized decision-making guided by clinical judgment, symptom burden and patient preference [46,47]. This approach consisted in identifying motivated patients with a good prognosis (no risk of renal progression) or those with many co-morbid conditions giving more importance to CKD compared to risk of death. This could probably explain that MMSE and QoL scores were not linked to renal prognosis in the OPAIR study. We can suppose that patients with the lowest QoL scores had no regular follow-up care and were withdrawn from dialysis. Previous data showed that an individualized model may be more appropriate than a disease-oriented model of care for many older adults with CKD [37,46-49]. Such individualized approach calls for listening to the patient, and providing prognosis and treatment information to patients and/or families. In particular, they should be informed that QoL is better improved in autonomous CKD patients on self-care dialysis than in those non-autonomous [50]. The patient will have anyway the final decision to be admitted or not into a dialysis program.

Our study has several limitations. First, the number of included patients was lower than expected. This highlighted the difficulty to establish a regular nephrology care in very elderly. As nephrology consultations are overloaded, elderly CKD patients are often followed-up by GPs. As a result, the analysis of risk factors for initiation of dialysis was underpowered. Indeed, only 17 dialysis initiations were observed during the study. For a multivariate analysis in survival analysis, it is recommended to have at least 10 events observed for each covariate [51]. Additionally, the risk of death was 2.5-fold greater than the risk of initiation of dialysis in our study. According to the incidence of dialysis initiation and death events in our study, it would be necessary to include at least 1,000 patients to study 10 covariates. Increasing the number of included patients and consequently the number of patients who initiated dialysis would require the extension of the enrolment period beyond 12 months. However, such decision would require a complex organization given the age of our patients. Second, the choice of both prevalent and incident CKD cases to be included into the study may have introduced a selection bias with an overrepresentation of prevalent cases with low risk of death as they had survived until study initiation. However, the study initiation started with the inclusion of patient to avoid the immortality bias. Third, we used the MDRD’s formula whereas it has not been yet validated in elderly. Additionally, as a function of both residual GFR and changes in lean body muscle mass (sarcopaenia) perhaps related to inflammation and chronic acidosis in stage 5 CKD, eGFR (MDRS’ formula) overestimates true GFR (inulin clearance) by about 3.3 mL/min/1.73 m^2^ or by about 42% [52]. Given the dependence of serum creatinine on muscle mass, there is a particular concern that eGFR slope in the elderly may be affected by changes in muscle over time. However, although potentially inaccurate as indicator of true GFR, eGFR is widely used in clinical practice and does seem to have prognostic value for death and ESRD [51]. Fourth, despite collecting extensive information about cognitive function and QoL, no positive association was drawn about their role as predictors of dialysis likelihood, on condition of no bias related to the small size of population.

## Conclusions

In summary, the consistent findings of our study provide an important extension of the contemporary literature on a comprehensive view of the burden of CKD and the common practice to offer dialysis in a pre-ESRD population of Octogenarians and Nonagenarians newly referred to nephrologists.

The reasons underlying dialysis decision-making were explored from GPs and nephrologists’ perspectives. We have shown that French nephrologists have a precise clinical judgement analysis in their dialysis decision-making based on uremia criteria; these findings confirm that nephrology referral is pertinent even in very elderly pre-ESRD patients.

This original competing-risk approach potentially highlights the appropriate targets and strategies for dialysis decision-making in very elderly healthy CKD patients. Nevertheless, OPAIR raises the question of the indication of dialysis in patients with cardiac failure. This clinical condition was an important cause of death.

Additionally, further clinical research including Octogenarians reaching end-stage renal failure is needed to determine the most appropriate treatment, conservative or renal replacement therapy, which should be offered regarding co-morbidities and health status. The ongoing follow-up of the large French PSPA cohort including 581 patients older than 75 years with end-stage renal failure (eGFR less than 20 mL/min/1.73 m^2^) should provide tools to help the physicians, the patients and their families in dialysis decision making process [53].

## Abbreviations

CI: Confidence interval; CKD: Chronic kidney disease; CRP: C-reactive protein; DBP: diastolic blood pressure; eGFR: Estimated glomerular filtration rate; ESRD: End-stage renal disease; Hb: Haemoglobin; MDRD’s formula: Modified diet in renal disease; MMSE: Mini Mental State examination; QoL: Quality of life; SD: Standard deviation; SF-36: Medical Outcomes Study 36-item Short-Form Health Survey; TSAT: Transferring saturation

## Competing interests

The authors declare that they have no competing interests.

## Authors’ contributions

Responsibility for the design, analysis, interpretation of data and conclusion lies with the authors. All authors gave their final approval regarding submission for publication. All authors read and approved the final manuscript.

## Authors’ information

BF, Head of Department of Nephrology, Hôpital Louis Pasteur, Colmar, France JBB, Research fellow of Department of Biostatistics, EA 2694, UDSL, Lille, France; Geriatric Department, University Hospital, Lille, France LF, Head of Department of Nephrology, University Hospital, Vandœuvre-lès-Nancy, France; EA 4360 Apemac, Nancy University, P. Verlaine Metz University, and Paris Descartes University, France.

## Pre-publication history

The pre-publication history for this paper can be accessed here:

http://www.biomedcentral.com/1471-2369/14/103/prepub
